# Developing an initial set of quality indicators for chiropractic care: a scoping review

**DOI:** 10.1186/s12913-024-10561-8

**Published:** 2024-01-12

**Authors:** Robert Vining, Jennifer Smith, Brian Anderson, Zachary Almquist, Danveshka Wong

**Affiliations:** 1 Palmer Center for Chiropractic Research, Palmer College of Chiropractic, 1000 Brady St, Davenport, IA USA; 2 Palmer College of Chiropractic, 1000 Brady St, Davenport, IA USA

**Keywords:** Chiropractic, Quality, Quality indicator, Benchmarking, Health care quality, Scoping review, Outcome and process assessment

## Abstract

**Background:**

Quality indicators are standardized, evidence-based measures of health care quality. Currently, there is no basic set of quality indicators for chiropractic care published in peer-reviewed literature. The goal of this research is to develop a preliminary set of quality indicators, measurable with administrative data.

**Methods:**

We conducted a scoping review searching PubMed/MEDLINE, CINAHL, and Index to Chiropractic Literature databases. Eligible articles were published after 2011, in English, developing/reporting best practices and clinical guidelines specifically developed for, or directly applicable to, chiropractic care. Eligible non-peer-reviewed sources such as quality measures published by the Centers for Medicare and Medicaid Services and the Royal College of Chiropractors quality standards were also included. Following a stepwise eligibility determination process, data abstraction identified specific statements from included sources that can conceivably be measured with administrative data. Once identified, statements were transformed into potential indicators by: 1) Generating a brief title and description; 2) Documenting a source; 3) Developing a metric; and 4) Assigning a Donabedian category (structure, process, outcome). Draft indicators then traversed a 5-step assessment: 1) Describes a narrowly defined structure, process, or outcome; 2) Quantitative data can conceivably be available; 3) Performance is achievable; 4) Metric is relevant; 5) Data are obtainable within reasonable time limits. Indicators meeting all criteria were included in the final set.

**Results:**

Literature searching revealed 2562 articles. After removing duplicates and conducting eligibility determination, 18 remained. Most were clinical guidelines (*n* = 10) and best practice recommendations (*n* = 6), with 1 consensus and 1 clinical standards development study. Data abstraction and transformation produced 204 draft quality indicators. Of those, 57 did not meet 1 or more assessment criteria. After removing duplicates, 70 distinct indicators remained. Most indicators matched the Donabedian category of process (*n* = 35), with 31 structure and 4 outcome indicators. No sources were identified to support indicator development from patient perspectives.

**Conclusions:**

This article proposes a preliminary set of 70 quality indicators for chiropractic care, theoretically measurable with administrative data and largely obtained from electronic health records. Future research should assess feasibility, achieve stakeholder consensus, develop additional indicators including those considering patient perspectives, and study relationships with clinical outcomes.

**Trial registration:**

Open Science Framework, https://osf.io/t7kgm

**Supplementary Information:**

The online version contains supplementary material available at 10.1186/s12913-024-10561-8.

## Background

The Agency for Healthcare Research and Quality (AHRQ) defines quality as “*the degree to which health care services for individuals and populations increase the likelihood of desired health outcomes and are consistent with current professional knowledge*” [1]. Formal attempts to improve quality occurred at least as early as the 1800s with Florence Nightingale, who strove to improve clinical outcomes by challenging contemporary practices, encouraging critical thinking, and promoting standardized processes thought to positively influence care [2]. In the late 20^th^ century, Avedis Donabedian proposed a systematic framework for assessing health care quality using quantitative measures, referred to as quality indicators. Donabedian’s framework describes indicators matching 3 major categories: Structure, Process, and Outcome [3].

Structural indicators describe the attributes of a setting where care occurs. Attributes include physical facilities, clinical equipment, organizational policies, and human resources. Process indicators refer to the steps taken to provide care such as examination, treatment, care planning, and scheduling. Outcome indicators describe the effects of care on patients and populations, such as short and long-term clinical improvement, satisfaction, and costs [4, 5]. The goal of quality assessment is to improve clinical outcomes. Structural indicators are fundamental to supporting care delivery (process), which in turn, influence outcomes.

In 2001, the Institute of Medicine (IOM) published a seminal report describing quality indicators as measurable elements of health care developed from scientific evidence, standards of practice and expert opinions that contribute to high-quality care. The 6 domains recommended in the IOM report as most relevant to health care quality include: 1. Safe; 2. Effective; 3. Patient-centered; 4. Timely; 5. Efficient; and 6. Equitable [3]. IOM domains reflect the most important aspects of health care that quality indicators should improve or maintain. Donabedian categories organize indicators according to their application. Both IOM domains and Donabedian categories are distinct, yet complementary, frameworks for classifying and developing quality indicators.

Historically, quality indicators were developed to measure hospital quality performance, which is evident in the definition still used by the Agency for Healthcare Research and Quality: *“standardized, evidence-based measures of health care quality that can be used with readily available hospital inpatient administrative data to measure and track clinical performance and outcomes”* [6]. However, quality indicators are no longer confined to in-patient hospital settings. A variety of healthcare disciplines and settings have developed, and continue to develop, quality indicators. For example, the Joint Commission uses quality indicators to assess and accredit home health services, nursing care centers, behavioral healthcare, ambulatory care centers and laboratory services [7]. Individual professions and specialty groups within professions have also developed quality indicators [8–11].

Chiropractic is a health profession focused primarily on nonpharmacological care for musculoskeletal conditions, with special emphasis on the spine and related conditions [12–14]. Chiropractic professionals function in both private, public, and multidisciplinary practice settings [12, 15, 16]. As a health profession, chiropractic carries an ethical obligation to conduct a variety of continuous learning activities directed toward improving the quality of clinical care [17]. However, without objectively measuring key aspects of care relating to quality, systematic quality improvement activities cannot be evidence-informed. Currently, there is no standard set of quality indicators for chiropractic care published in peer reviewed literature.

Stelfox and colleagues recommend conducting a multi-step process for developing and validating quality indicators [18, 19]. The first step is conducting a systematic literature review to identify best practices and other evidence to support draft indicators obtainable from administrative data. A variety of potential validation processes should follow, using consensus and other research methods. The long-term goal of this line of research is to develop and validate a set of quality indicators for chiropractic care. The objective of this study is to identify current professional knowledge from clinical guidelines, best practice publications, and professional standards to:

A) develop a preliminary set of quality indicators for chiropractic care, measurable with administrative data without the need for individual file audits;
B) identify gaps and opportunities for additional quality indicator development; and
C) inform future research directions for subsequent refinement and validation.

## Methods

We conducted a scoping review because: 1) there was a need for systematic literature search methods designed to closely examine a topic on which limited and/or disparate knowledge exists, to identify gaps, and to systematically organize information to direct further research [20, 21]; 2) the source literature in this study was known to include non-peer reviewed sources [22]; 3) the study objectives addressed questions beyond those about effectiveness of interventions, focused instead on transforming recommendations into potential quantitative measures [21]; 4) critical appraisal of included sources was not required [23]; and 5) transparent reporting of data synthesis methods was vital [23].

This scoping review followed PRISMA guidelines (Preferred Reporting Items for Systematic Reviews and Meta-Analyses) for scoping reviews (PRISMA-ScR), prospectively registered with Open Science Framework on 30 August, 2022 (https://doi.org/10.17605/OSF.IO/T7KGM) [24]. Consistent with recommendations for developing quality indicators, we used a deductive approach to identify evidence-based concepts and recommendations from clinical guidelines, best practice publications, and quality standards [3, 18]. Once identified, we transformed these findings into more specific and measurable quality indicators consistent with the frameworks proposed by Donabedian and the IOM [3, 19].

### Search strategy

A systematic literature search was conducted by a health sciences librarian (JS) on August 31, 2022 of PubMed/MEDLINE, CINAHL (EBSCOhost interface), and Index to Chiropractic Literature databases. Results were restricted to English language studies published between January 1, 2012-August 31, 2022. Search terms consisted of subject headings specific to each database and free text words related to chiropractic, musculoskeletal pain, and quality indicators. The complete search strategies for each database are available as Supplementary file 1. The search was validated using a sample of 24 articles identified by the authors as potentially eligible and therefore should appear in search results (Supplementary file 2). General internet search engines were also used to explore potential quality indicators or other quality standards not otherwise available in peer reviewed literature. An updated search was performed on April 19, 2023 to account for potential articles published during the eligibility determination and data abstraction and transformation stage of this study. Reference searching was not employed because we only included the most recent versions of source documents.

### Eligibility criteria

Because care standards, best practices, and clinical guidelines are designed to adapt as new evidence emerges, we limited our article eligibility to 10-years from our original search (2012-present) [25]. Eligible articles were written in English, measured an aspect of chiropractic care quality, and developed best practices or clinical guidelines directly applicable to chiropractic care. Non-peer-reviewed literature sources were eligible when quality indicators or quality standards pertaining to chiropractic care were included, such as quality measures published by the Centers for Medicare and Medicaid Services, quality standards published by Royal College of Chiropractors, U.K., and low back pain clinical care standards published by the Australian Commission on Safety and Quality in Health Care [26–28].

Ineligible articles included those that did not explicitly develop quality indicators for chiropractic care, studies reporting on the efficacy or effectiveness of interventions, guideline reviews, guidelines for other health disciplines, and epidemiological research. Best practice and guideline documents for which an updated publication was available were also ineligible. Articles reporting on studies conducted with animals, tissues, or cadaveric specimens, conference proceedings or abstracts, editorials, commentaries, articles recommending care practices based on narrative reviews, and case reports or case series, were also ineligible.

Article eligibility was assessed by 2 authors (BA, DW) in sequential steps beginning with article titles, followed by abstract review, then full text review of remaining articles. Ineligible articles were removed at each stage. Discrepancies were resolved through consensus discussion among both reviewers. When eligibility was unclear, the lead investigator (RV) rendered the final determination.

### Data abstraction

Primary data abstraction was performed independently by 2 authors (RV, BA), with over 45 years of combined chiropractic clinical and research experience. A data abstraction form facilitated this process, which included the categories for abstracting the evidence source, condition addressed, title of potential indicator, description, corresponding Donabedian category and IOM domain(s), evidence level, and metric. Data abstraction involved identifying specific statements within the included literature that may conceivably be measured. Once identified, the statements were recorded on the data abstraction form, initiating the transformation process.

### Quality indicator transformation

**Fig. 1:**
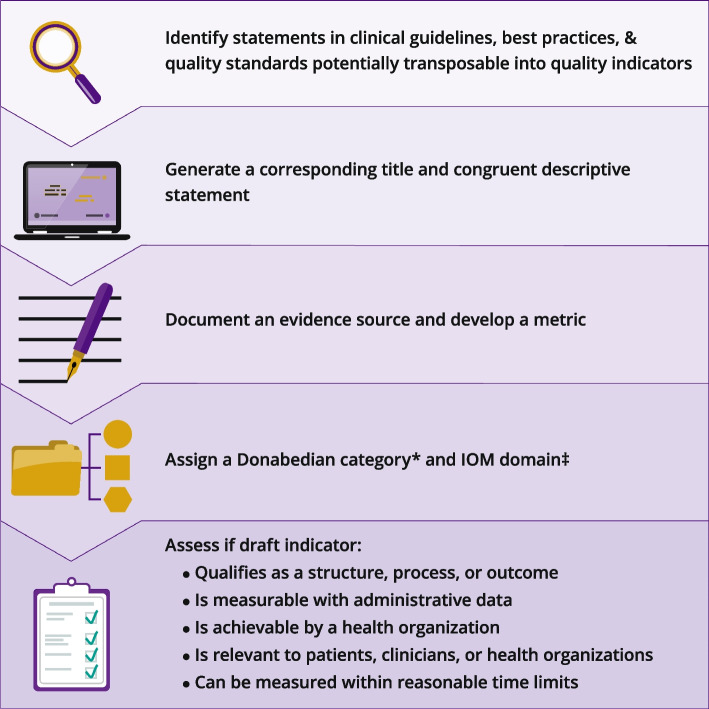
Graphic depiction of the quality indicator abstraction and transformation process. *Donabedian categories: Structure (attributes of a setting where care occurs such as physical facilities, clinical equipment, policies, and human resources); Process (measurable activities performed to provide care, such as examination and treatment); Outcomes (measurable effects of care on patients and populations); ‡: Institute of Medicine, now referred to as the National Academy of Medicine, United States

Quality indicator development lacks transparent methodological reporting for some healthcare disciplines [29]. We adopted a stepwise transformation process to review included literature and transform statements and recommendations into quality indicators (Fig. 1). The process included:

1. Generating a brief title and descriptive statement
2. Developing a Metric (e.g., policy, human or physical infrastructure description, or numerator and denominator) and documenting an evidence source
3. Assigning a primary Donabedian category and relevant IOM domain
4. Assessing potential quality indicators according to the following criteria [30]:
  Describes a narrowly defined structure, process, or outcome while also matching 1 or more IOM domains: safe; effective; patient-centered; timely; efficient; equitable
  Quantitative data can conceivably be available to measure the potential indicator
  The performance designated is achievable by a health organization or clinician
  The metric is relevant to those involved, such as patients, family members, clinicians, or health organizations
  Data can be collected in aggregate within reasonable time limits
5. Assigning an evidence level consistent with the Oxford Centre for Evidence-Based Medicine model (March 2009) [31].

The transformation process included the following principles:

1. Statements requiring individual file audit or clinical judgment were not transformed. (e.g., providing evidence-based care, management of comorbidities), which require consideration of multiple elements of the clinical record such as the health history, problem severity, patient preferences, and treatment response.
  - When it was unclear if statements from source documents were transformable into measurable indicators, a draft was attempted and later evaluated with the assessment criteria.
2. Recommendations for elective interventions or those dependent on patient consent or preference were not transformed because such actions are optional for providers and/or patients.
3. Statements, standards, and recommendations to avoid specific activities (e.g., routine imaging for acute low back pain) were not transformed because individual case-level review is needed to assess clinical reasoning and determine appropriateness.
4. Statements, recommendations, and standards focused on specific conditions or presentations (e.g., neck pain, headache, pregnancy) were transformed into generalized indicators when they applied universally (e.g., Informed consent, Examination, Red flag screening).
5. Though some indicators can potentially relate to multiple IOM domains, only the domain judged most relevant was assigned.
6. Descriptions and metrics for some indicators, such as those derived from the Royal College of Chiropractors and Centers for Medicare and Medicaid, were revised for consistent formatting.
7. Comparable (i.e., redundant) indicators were combined into single indicators.

After initial data abstraction and transformation, authors (JS, ZA, DW) used a standardized checklist (Supplementary file 3) to guide critical review of each transformed potential indicator.

While we initially reported evidence levels, it became apparent that most indicators were rated with an evidence level of 5 (expert opinion or based on physiology, bench research, or first principles). Conducting separate literature reviews to confirm the accuracy of these ratings was beyond the scope of this project. Therefore, evidence rating was discontinued to avoid potential misreporting.

## Results

**Fig. 2:**
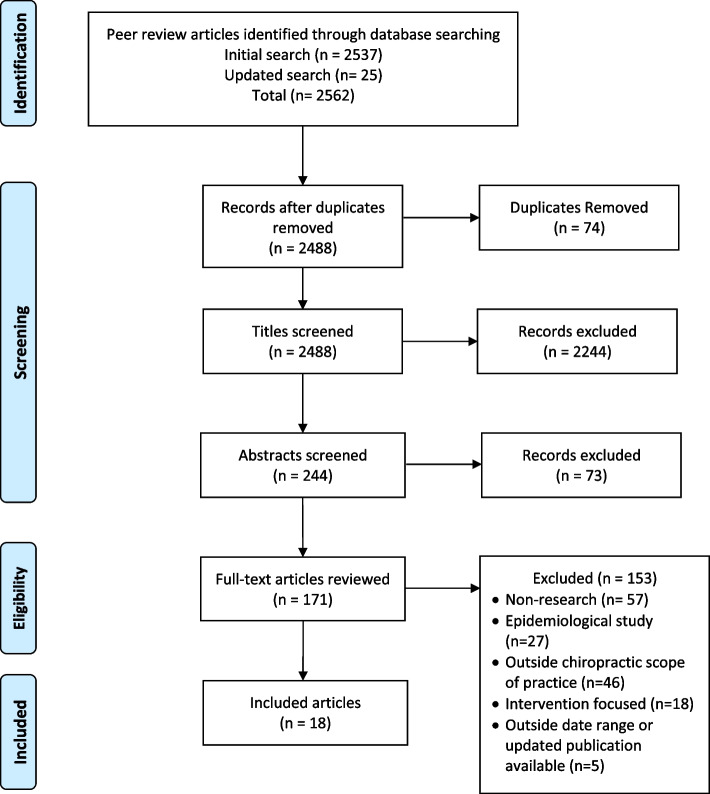
PRISMA flow diagram

The original literature search revealed 2562 articles. A second updated search identified an additional 25 articles. After removing duplicates, 2488 articles remained. Most of the 18 articles meeting final eligibility criteria were clinical guidelines (*n* = 10) [32–41]. The remaining articles consisted of best practice recommendations (*n* = 6) [42–47], a modified Delphi study (*n* = 1) [48], and a clinical appropriateness standards development study (*n* = 1) [49]. Figure 2 summarizes the search and eligibility determination process consistent with PRISMA recommendations. We also identified non-peer-reviewed sources meeting eligibility criteria: a clinical guideline from U.S. Veteran’s Health Affairs/Department of Defense, quality standards from the Royal College of Chiropractors (U.K.), quality measures from the Centers for Medicare and Medicaid Services, and low back pain standards published by the Australian Commission on Safety and Quality in Health Care [26–28, 50].

A total of 204 quality indicators were abstracted and transformed from included sources. Of those, 57 did not meet 1 or more criteria for specificity, measurement with administrative data, practicality, relevance, or timely data collection. The remaining 147 were then sorted by topic area. After combining redundant indicators, 70 unique items remained.

The largest number of indicators developed in this study match the Donabedian category of process (*n* = 35). These indicators were developed from statements within 19 different included sources. Most indicators relating to organizational structure (*n* = 31) were derived from quality standards published by the Royal College of Chiropractors (U.K.) [27]. Only 4 indicators match the Donabedian category of outcome. IOM domains from most to least common included: Effective (*n* = 25), Safe (*n* = 21), Patient-Centered (*n* = 16), Efficient (*n* = 5), Timely (*n* = 2), and Equitable (*n* = 1).

Table 1 displays titles, descriptions, and metrics for quality indicators matching the Donabedian category of structure. Table 2 displays process-related indicators, and Table 3 displays indicators related to outcomes of care.

**Table 1:** Quality indicators related to organizational structure

**Title**	**Source**	**Type**	**Description**	**IOM Domain**	**Metric**
Activity advice policy	[27]	Low back pain	The organization supports advice to stay active for patients with low back pain	Effective	Policy requiring providers to advise patients with low back pain to stay active
Appointment wait time policy	[27]	General	The organization supports scheduling chiropractic care within 3 working days after time of request	Timely	Policy designating acceptable wait times for chiropractic care
Appointment wait time scheduling capacity	[27]	General	Resource capacity adequate to offer patients an appointment within 3 working days	Timely	Documented human and facilities resources demonstrating capacity to achieve designated performance (e.g., evidence of # of providers, # of rooms available for evaluation/treatment, # of appointments available per day/week/month)
Audit policy	[27]	General	The organization recognizes the need for regular audits to ensure compliance with regulatory bodies, identify shortcomings, etc	Efficient	Policy designating periodic quality control audits
Audit process	[27]	General	The organization conducts regular audits	Efficient	Results of completed audits
Care plan policy	[27]	General	The organization supports multimodal chiropractic care	Patient-Centered	Policy requiring care plans with designated components
Continuity of care policy	[27]	General	The organization supports continuity of care with the same provider	Patient-Centered	Policy and infrastructure supporting care delivered to patients by the same provider
Cost transparency	[48]	General	Costs of chiropractic care are transparent and facilitate access	Equitable	Evidence of public access to costs of initial and follow-up chiropractic visits, or costs for chiropractic services
Diagnostic imaging policy	[27, 28, 37]	General	The organization has policy and practical resources designating indications for diagnostic imaging consistent with clinical guidelines or relevant best practices	Efficient	Policy and practical resources to guide diagnostic imaging use
Examination policy	[27]	General	The organization expects examination of a body region prior to delivering care to the area	Safe	Policy directing clinical evaluation prior to engaging in clinical interventions
Explanation policy	[27]	General	The organization expects providers to communicate an explanation of the suspected/confirmed condition to patients	Patient-Centered	Policy requiring providers to communicate an explanation of their suspected/confirmed condition
Health and condition specific history policy	[27]	General	The organization requires obtaining health and condition specific history prior to clinical evaluation and management	Effective	Policy requiring a documented health and condition specific history for a problem being evaluated/managed
Infection control protocols	[45]	General	The organization supports infection control and prevention through established protocols	Safe	Documentation of infection control protocols
Infection control training	[45]	General	Training procedures are in place, such as hand hygiene, personal protective equipment, and environmental cleaning	Safe	Documentation of hygienic training procedures
Informed consent policy	[27]	General	The organization requires informed consent from a patient, parent, or legal guardian prior to delivering clinical services	Patient-Centered	Policy requiring informed consent from patients, parents, or legal guardians, prior to delivering clinical services
Interprofessional collaboration policy	[37]	General	The organization recognizes the responsibility of providers to refer, co-manage, and/or communicate with other healthcare professionals when clinically indicated and authorized by patients	Effective	Policy recognizing the need to collaborate with other healthcare professionals
Mind-body resources	[45]	Spine-related chronic pain and osteoarthritis	Resources are available for mind-body interventions such as Cognitive-Behavioral Therapy and Mindfulness-Based Stress Reduction	Patient-Centered	Documentation of patient-accessible mind-body resources
Organizational planning	[27]	General	The organization has employment and training procedures, a reporting/supervisory structure, and a future planning strategy	Efficient	Documentation of employment and training procedures, organizational structure, and future planning strategy
Osteoporosis risk factor screening policy	[27]	Osteoporosis	The organization supports screening patients for major risk factors for osteoporosis	Safe	Policy designating screening patients over age 40 for major risk factors for developing osteoporosis
Patient experience data collection	[27]	General	The organization collects data from patients about their care	Patient-Centered	Data from patients about the experience of receiving care
Provider credential database	[27]	General	The organization maintains a database of professional credentials for all providers	Effective	Database documenting current professional credentials of all providers
Psychosocial factor intervention infrastructure	[27, 28]	General	The organization maintains accessible resources to support cognitive and behavioral interventions, and connecting patients with potentially helpful social resources	Effective	Evidence of referral pathways and other community resources to support psychological and social health
Psychosocial factor screening policy	[27, 28]	General	The organization supports screening for relevant psychosocial factors	Effective	Policy designating early psychosocial screening
Records management policy (privacy)	[27]	General	The organization secures patient information according to regulatory requirements	Patient-Centered	Policy designating compliance with regulatory requirements for health record privacy and security
Re-evaluation Policy	[27]	General	Regular monitoring and re-evaluation is required to facilitate decisions for discharge, continued care, or referral	Patient-Centered	Policy requiring regular re-evaluation of ongoing care to inform decisions for discharge, continued care, or referral
Red flag screening policy	[27, 28]	General	Screening for serious underlying pathology is required by the organization	Safe	Policy requiring screening for serious underlying pathology
Referral system	[28, 45]	General	A referral system is in place for patients with signs and/or symptoms of conditions outside the scope of chiropractic care	Safe	Documented referral pathways for patients needing services from specialists
Safety and risk management policy	[27]	General	The organization prioritizes safety through incident reporting, clinical risk management, and adverse event reporting	Safe	Policy and procedures to support a culture of safety including incident reporting, clinical risk management, and adverse event reporting
Self-management policy	[27]	General	The organization encourages interventions to support patient self-management capacity such as education and active care approaches	Patient-Centered	Policy supporting improving self-management capacity among patients
Shared decision-making policy	[27]	General	The organization supports shared decision-making among providers and patients	Patient-Centered	Policy requiring providers to engage in shared decision-making in the care planning process
Suicide prevention pathway	[45]	General	A referral system is in place for patients with increased risk for suicide	Safe	Documented referral pathway for patients with increased suicide risk

**Table 2:** Quality indicators related to clinical processes

**Title**	**Source**	**Type**	**Description**	**IOM Domain**	**Metric**
Additional care assessment	[35, 36, 40, 41, 47]	General	Percentage of visits where the need for additional care is assessed	Efficient	Numerator: # of follow-up visits documenting an assessment for additional care; Denominator: # of follow-up visits
Body Mass Index (BMI) screening	[26, 45]	General	Percentage of patients screened for abnormal BMI	Effective	Numerator: Patients with a documented BMI; Denominator: All patients
Cognitive health screening	[43]	Older adults	Percentage of older adults screened for cognitive health status	Safe	Numerator: # of older adults screened for cognitive health status; Denominator: # of older adults
Continuity of care	[27]	General	Percentage of patients receiving care from the same provider	Patient-Centered	Numerator: # of patients receiving care from the same provider; Denominator: # of patients receiving care
Current Care plans	[27, 47]	General	Percentage of visits (or patients) with an unexpired care plan	Safe	Numerator: # of visits (or patients) with a corresponding unexpired care plan; Denominator: # of visits (or patients)
Current Medication list	[26]	General	Percentage of patients with a documented list of current medications	Safe	Numerator: # of patients with a current list of medications in the clinical record; Denominator: # of patients
Diagnostic triage	[49]	Low back pain	Percentage of patients with low back pain allocated into one or more categories: specific pathology; radicular syndrome; non-specific; other (e.g., low back pain from visceral sources)	Effective	Numerator: # of new consultations for low back pain with a documented diagnostic category; Denominator: total # of new consultations for low back pain
Dietary advice	[45]	General	Percentage of patients provided with dietary advice emphasizing items such as vegetables, fruit, and unprocessed foods	Effective	Numerator: # of patients provided with general dietary advice; Denominator: # of patients
Examination	[27, 40, 43, 44, 47]	General	Percentage of patients who receive a clinical examination for a presenting problem	Safe	Numerator: # of visits for a new problem with a corresponding examination for the problem; Denominator: # of visits for a new problem
Fall prevention (Older adults)	[45]	Older adults	Percentage of older adults offered advice on balance, strength, and endurance exercises to prevent falls	Safe	Numerator: # of older adult patients offered advice on exercises to prevent falls; Denominator: # of older adult patients
Functional health screening (Older adults)	[43]	Older adults	Percentage of older adults screened for abilities to independently carry out activities of daily living	Safe	Numerator: # of older adults screened for functional health status; Denominator: # of older adults
General health screening	[43]	General	Percentage of patients screened for general health status	Safe	Numerator: # of visits for a new problem documenting general health status; Denominator: # of visits with a new problem
Health and condition specific history	[27, 42–44, 46, 47, 49]	General	Percentage of patients with documented past health and condition specific history prior to initiating care	Patient-Centered	Numerator: # of visits for a new problem documenting designated components of a history; Denominator: # of visits for a new problem
Home-environmental safety screening (Older adults)	[43]	Older adults	Percentage of older adults screened for home environmental safety	Safe	Numerator: # of older adults screened for home-environment safety; Denominator: # of older adults
Hypertension screening	[26]	Hypertension	Percentage of patients screened for hypertension	Effective	Numerator: # of patients screened for hypertension; Denominator: # of patients
Informed consent process	[27, 40, 42–47]	General	Percentage of patients receiving care only after completing an informed consent process	Patient-Centered	Numerator: # of care plans documenting informed consent from patient, parent, or guardian; Denominator: # of care plans
Multimodal care plans	[27, 28, 32–34, 40, 41, 44, 47, 49]	General	Percentage of care plans including 5 components: 1) Active therapies such supervised or unsupervised exercise; 2) Manual therapies such as joint manipulation, and myofascial therapies; 3) Education about one’s condition including pain physiology when appropriate; 4) Self-management advice and/or activities; and 5) Therapeutic goals. *Multimodal interventions are not required at each visit during a care plan	Effective	Numerator: # of care plans including Active therapies; Manual therapies; Education; Self-management advice and/or activities; Goals; Denominator: # of care plans
Neck pain classification	[35]	Neck pain	Percentage of patients with neck pain classified as Grade I, II, III, or IV	Effective	Numerator: # of patients with neck pain and a Grade I-IV classification; Denominator: # of visits recording new neck pain
Nutritional health status screening	[43]	General	Percentage of patients screened for nutritional health status	Effective	Numerator: # of patients screened for nutritional health status; Denominator: # of patients
Opioid use screening	[45]	General	Percentage of patients screened for current opioid use	Effective	Numerator: # of patients screened for current opioid use; Denominator: # of patients
Osteoporosis referral	[27]	Osteoporosis	Percentage of patients at risk for osteoporotic fracture referred to a primary care provider	Effective	Numerator: # of patients with significant risk for osteoporotic fracture referred to a primary care provider; Denominator: # of patients with significant risk for osteoporotic fracture
Osteoporosis risk factor screening	[27]	Osteoporosis	Percentage of patients over age 40 screened for major risk factors for osteoporosis	Safe	Numerator: # of patients over age 40 assessed for major risk factors for osteoporosis; Denominator; # of patients over age 40
Osteoporotic fracture referral	[27]	Osteoporosis	Percentage of patients with new/recent osteoporotic fracture referred to a primary care or other relevant provider	Effective	Numerator: # of patients with new/recent osteoporotic fracture referred to a primary care or other relevant provider; Denominator: # of patients with new/recent osteoporotic fracture
Outcome assessment (baseline)	[40]	General	Percentage of patients assessed with valid functional and/or symptom outcome measures	Effective	Numerator: # of visits for a new problem that include a valid baseline outcome assessment; Denominator: # of visits for a new problem
Pain classification	[38]	General	Percentage of patients with pain with a working diagnosis of nociceptive, neuropathic, and/or nociplastic pain	Effective	Numerator: # of patients with pain with documented working diagnosis of nociceptive, neuropathic, and/or nociplastic pain; Denominator: # of patients with pain
Physical activity screening	[45]	General	Percentage of patients screened for physical activity level	Effective	Numerator: # of patient visits for a new problem with physical activity screening; Denominator: # of visits with a new problem
Psychosocial factor screening	[27, 35, 44, 47, 49, 50]	General	Percentage of patients assessed for psychological and social risk factors for poor outcome and/or chronicity	Effective	Numerator: # of visits for a new problem with psychosocial screening; Denominator: # of visits with a new problem
Radiographic screening	[46]	General	Percentage of patients screened for the possibility of pregnancy prior to obtaining radiographs	Safe	Numerator: # of radiographic exams where the possibility of pregnancy is documented; Denominator: total # of radiographic exams
Re-evaluation	[27, 40]	General	Percentage of follow-up care plans based on a clinical re-evaluation	Effective	Numerator: # of follow-up care plans based on a clinical re-evaluation; Denominator: # of follow-up care plans
Red flag screening	[27, 28, 36, 40–42, 46, 47, 50]	General	Percentage of patients screened for signs and symptoms of serious pathology	Safe	Numerator: # of visits for a new problem with screening for serious underlying pathology; Denominator: # of visits for a new problem
Response to care	[27]	General	Percentage of patients whose response to care is regularly assessed	Effective	Numerator: # of follow-up visits documenting response to prior care; Denominator: # of follow-up visits in a care plan
Review of systems	[42]	General	Percentage of patients whose clinical record includes a review of systems such as cardiovascular, pulmonary, integumentary, etc	Safe	Numerator: # of patients with a documented review of systems; Denominator: # of patients
Shared decision-making process	[36, 41, 49]	General	Percentage of patients involved in care planning and decision-making	Patient-Centered	Numerator: # of patients involved in care planning and decision-making; Denominator: # of patients with care plans
Tobacco use Screening	[26, 45]	Tobacco use	Percentage of patients screened for tobacco use	Effective	Numerator (criterion 1): # of patients screened for tobacco use; Denominator: # of patients
Vital signs	[43, 46]	General	Percentage of patients whose vital signs are recorded	Safe	Numerator: # of visits for a new problem with documented vital signs; Denominator: # of visits for a new problem

**Table 3:** Quality indicators related to outcomes of care

**Title**	**Source**	**Type**	**Description**	**IOM Domain**	**Metric**
Outcome assessment (re-evaluation)	[26, 27, 40, 44, 47]	General	Percentage of patients assessed during a re-evaluation using a valid functional and/or symptom outcome measure	Effective	Numerator: # of re-evaluation visits including a valid outcome assessment instrument; Denominator: # of re-evaluation visits
Patient satisfaction	[48]	General	Percentage of patients reporting satisfaction with chiropractic care	Patient-Centered	Numerator: # of patients reporting satisfaction with care; Denominator: # of patients responding to questions about satisfaction
Return to work	[48]	General	Percentage of patients unable to work due to a work-related injury returning to work in a timely manner	Effective	Numerator: # of non-working patients treated for a work-related injury who returned to work within designated timeframes (e.g., 30 days, 60 days, 90 days); Denominator: # of non-working patients treated for a work-related injury
Shared decision-making outcome	[36]	General	Percentage of patients reporting involvement in care planning and decision-making	Patient-Centered	Numerator: # of patient surveys reporting shared decision-making was part of the care planning process; Denominator: # of patient surveys

## Discussion

To the authors’ knowledge, this is the first study to propose an initial set of quality indicators using scoping review methodology and a transparent process for abstracting and transforming data from recent clinical guidelines, best practice publications, and quality standards for chiropractic care. Quality standards and quality indicators share some similar characteristics. The Royal College of Chiropractors quality standards describe chiropractic care ideals while offering sample metrics, several of which are measurable through individual file audits [27]. Alternatively, this project developed indicators consistent with the definition from the Agency for Healthcare Research and Quality, which are derived largely from administrative data. Indicators obtained from administrative data offer quality assessment across a health organization while avoiding dependence on individual file audits and limitations related to inadequate sample size, and the lack of expertise and potential bias of auditors.

Angel-Garcia et al., reported 178 quality indicators for hospital-based physical therapy, several of which share similarities with those developed in this study, such as conducting an exam, obtaining informed consent, and depression screening [51]. Newell et al., demonstrated the feasibility of collecting patient reported outcomes from chiropractic patients using online survey methods [52]. More recently, Blanchette et al., proposed a set of indicators to evaluate chiropractic performance on a provincial or national scale in Canada and the Australian Commission on Safety and Quality in Health Care published low back pain clinical care standards largely applicable to chiropractic [28, 48]. This study is unique for the following reasons: 1) we used systematic and transparent literature search methods; 2) we focused on developing indicators for chiropractic care at the health organization level and measurable with administrative data; 3) we developed indicators consistent with the guiding frameworks described by Donabedian and the IOM; and 4) we proposed a preliminary set of indicators for subsequent refinement and validation.

### Practical considerations

Structural indicators are largely measurable through policies and documents describing a health organization, such as facilities, technical capacities, and mission [5, 53]. Most proposed process and outcome indicators are theoretically measurable with structured data contained in electronic health records, though modifying individual systems may be needed. Metrics developed in this study did not designate specific timeframes for each indicator, leaving those decisions to individual health organizations as they consider resources, goals, and other factors unique to each setting. The importance, value, and implementation of some indicators can depend on distinct characteristics of each health organization and patient population where chiropractic services are offered.

Quality indicators have historically been used in multi-provider settings. Therefore, the indicators developed in this study are likely most applicable to multi-provider organizations with the capacity to conduct ongoing quality assessment and improvement processes. Although most chiropractic care has historically been available from sole practitioners, there is a growing presence in multi-provider and multidisciplinary settings. Chiropractic services are now offered in hospital-based health systems, through corporate health organizations, and at U.S. military health treatment facilities, Olympic training centers, and Veterans Affairs facilities [54–57]. Given the increasing sophistication of electronic health records, it is conceivable that using quality indicators may also be feasible for individual providers.

Activities involved in delivering and recording health care are interrelated and complex, posing challenges for data collection and interpretation. If documenting quality indicator data impedes clinical flow, extends appointment durations, burdens provider documentation, distracts provider focus, or negatively impacts provider morale, there could be an unintended negative influence on the quality of care [58, 59]. Readers are encouraged to consider these practical factors when developing data collection methods, including what is most important for the setting, quality assessment timelines, impact on how services are delivered, and resources needed [60].

### Interpreting quality indicator data

There are several factors relevant to accurately interpreting data from quality indicators proposed in this study. First, quality indicators are individually measurable components associated with quality care. No single indicator represents a comprehensive assessment of quality. Accurate interpretation may require carefully assessing data from multiple indicators combined with context knowledge about health organization characteristics, clinical processes, populations served, and an understanding of how structure, process, and outcome indicators interrelate.

For example, shared decision-making is a central attribute of patient-centered care and a feature of quality [61]. To collect shared decision-making data from electronic health records, administrative support, technological capacity, and provider training are likely needed. Should these structural elements support systematic documentation in electronic health records, data would reflect how often providers engage in shared decision-making processes. However, engaging in a process does not guarantee a desired outcome. Patient generated data (e.g., surveys) are needed to determine if the clinical processes are effective.

Second, because this study sought to propose an initial set of indicators for chiropractic care, there was a concerted effort to include those thought to be theoretically attainable rather than only those known to be attainable (e.g., those previously measured and reported such as functional outcome measures). This methodological process helped maximize the number of preliminary indicators developed in this study while minimizing unintentional author bias by presuming that indicators could be measured when it was unclear if measurement was possible [62, 63]. Further, all indicators developed in this study may not be feasible for every health organization. Additional study is needed to refine and validate these findings and to develop potentially missing indicators.

Third, quality indicators were not developed to assess appropriate imaging use because imaging decisions are dependent on multiple factors unique to each patient and clinical scenario. Quality indicators are instead designed to be derived from administrative data, without the need for individual file review. Given the persistent challenge of unnecessary imaging in healthcare [64, 65], quality improvement programs may consider if limited file review in such areas is needed.

Fourth, this study did not detect sources specifically identifying recommendations, best practices, or clinical standards generated from patient perspectives. Additional research is needed to develop meaningful indicators informed by patients. Given the initial set of indicators developed in this study, a logical next step is to begin a validation process through expert review and consensus among various stakeholders such as patients, clinicians, health system administrators, and researchers [19].

### Multimodal chiropractic care plans

The sources included in this review consistently recommended multimodal chiropractic care regardless of patient population or condition. However, recommendations about multimodal care were described incongruently. For example, some clinical guideline recommendations focused primarily on specific interventions [34, 36]. Other source recommendations focused on whole person approaches, describing multimodal care in categorical terms (e.g., active care, passive care) [27, 44]. Condition-specific education was variably described, though routinely recommended as a fundamental component of care [27, 28, 32, 34, 35, 41, 47, 49]. The disparate nature of statements within source publications led to developing overlapping draft care plan indicators. To address this challenge, we developed an indicator representing a synthesis of recommendations which assess if care plans include:

- Active therapies such as supervised or unsupervised exercise;
- Manual therapies such as joint manipulation, mobilization, myofascial therapies, and passive muscle stretching;
- Education about one’s condition, including pain physiology when appropriate;
- Self-management advice and/or activities.
- Therapeutic goals

Structuring care plans to include these categories theoretically facilitates: 1) Care consistent with existing guidelines, best-practice recommendations, and quality standards; 2) Addressing biological, psychological, social, and environmental factors; 3) Freedom to construct care plans individually; 4) Education to help patients understand a problem and make more informed decisions; 5) Applied learning focused on reducing/preventing dependence on providers and supporting self-management capacity; and 6) Active patient engagement. The multimodal care plan approach may also support outcomes beyond pain reduction. For example, education, self-management activities, and active therapies may help improve condition specific health literacy and self-efficacy while personalized care and mutually agreed goals foster therapeutic alliance [66].

Because some elements may not be needed in individual circumstances, including treatments for each intervention category should not be mandatory in every care plan. However, it is possible to efficiently document a reason why a category was not included (e.g., patient declines). In addition, the source literature obtained in this study was oriented toward care for patients with singular pain-related conditions. Future study is needed to assess if the multimodal care plan indicator proposed in this study is feasible for non-pain-focused care, such as improving or maintaining physical function, when chiropractic care is part of an interdisciplinary care plan, or when addressing more than 1 problem [67–69].

### Limitations

Despite systematic search and eligibility determination methods, it is possible some relevant articles, including non-English publications and other non-peer reviewed sources, were missed. We used a data abstraction and transformation process including defined criteria and multiple levels of review to develop this initial set of quality indicators. Nevertheless, all indicators reported may not be measurable or necessarily contribute to health care quality in every setting where chiropractic services are available. Some overlap may exist among some indicators and data may not be obtainable in some settings due to missing or limited human and/or other infrastructure such as electronic health record systems.

Though systematic, the process of quality indicator development required human interpretation and judgment. Examples include transforming quality indicators generated from sources referencing specific conditions or patient groups (e.g., low back pain, neck pain, pediatric patients) into general indicators because the concepts were considered to apply universally (e.g., informed consent, red flag screening, multimodal care). We also combined redundant draft indicators, a process requiring human judgment. Finally, we did not assess the strength of evidence supporting each proposed indicator because performing secondary literature searches for each was beyond the scope of this project. Should the proposed quality indicators be adopted by health organizations, the data generated from their use can be used to further test, develop, and validate potential associations with clinical outcomes.

## Conclusions

This article proposes a preliminary set of 70 quality indicators for chiropractic care. Most fit Donabedian categories of process and structure, highlighting a need to develop additional outcome measures, especially those meaningful to patients. Few indicators developed in this study relate to IOM categories of Timely, Equitable, and Efficient. Future work should focus on refining and expanding this preliminary set by engaging with relevant stakeholders and assessing the feasibility of collecting and analyzing quality indicator data through quality improvement/assurance processes.

### Supplementary Information

**Additional file 1.** Search strategy.

**Additional file 2.** Search validation list.

**Additional file 3.** Quality indicator 2^nd^ level review checklist.

## Data Availability

Data analyzed in this study is included in the source publications used in this research.

## Abbreviations

IOM: Institute of Medicine
PRISMA: Preferred Reporting Items for Systematic Reviews and Meta-Analyses
PRISMA-ScR: Preferred Reporting Items for Systematic Reviews and Meta-Analyses for Scoping Reviews
