# Supplementary material for: Developing an initial set of quality indicators for chiropractic care: a scoping review
Source: BMC Health Serv Res. 2024 Jan 12;24:65. doi: 10.1186/s12913-024-10561-8 (PMC10785553; doi:10.1186/s12913-024-10561-8)
Supplement: Supplementary file 1 — Additional file 1. Search strategy. [file 12913_2024_10561_MOESM1_ESM.pdf]

Search strategy

PubMed

1. ("Spinal Stenosis"[MeSH Terms])
2. ("Musculoskeletal Pain"[Mesh:NoExp])
3. ("Shoulder Pain"[MeSH Terms])
4. ("Low Back Pain"[MeSH Terms])
5. ("Neck Pain"[MeSH Terms])
6. ("Migraine Disorders"[MeSH Terms])
7. ("Headache"[MeSH Terms:noexp])
8. ("Chiropractic"[MeSH Terms])
9. ("manipulation, chiropractic"[MeSH Terms])
10. ("manipulation, orthopedic"[MeSH Terms])
11. ("chiropractic"[Text Word])
12. ("chiropractor"[Text Word])
13. ("spine pain"[All Fields] OR "spinal pain"[All Fields])
14. #1 OR #2 OR #3 OR #4 OR #5 OR #6 OR #7 OR #8 OR #9 OR #10 OR #11 OR #12 OR #13
15. ("clinical practice guideline"[All Fields])
16. ("Practice Guideline"[Publication Type])
17. ("Practice Guidelines as Topic"[MeSH Terms])
18. ("Guideline"[Publication Type])
19. ("Guidelines as Topic"[MeSH Terms:noexp])
20. ("standards"[MeSH Subheading])
21. ("Quality of Health Care"[MeSH Terms:noexp])
22. ("quality assurance, health care"[MeSH Terms:noexp])
23. ("Benchmarking"[MeSH Terms])
24. ("quality indicators, health care"[MeSH Terms:noexp])
25. ("Quality Improvement"[MeSH Terms:noexp])
26. ("Standard of Care"[MeSH Terms:noexp])
27. ("Quality Control"[MeSH Terms:noexp])
28. ("best-practice recommendations"[Text Word])
29. #15 OR #16 OR #17 OR #18 OR #19 OR #20 OR #21 OR #22 OR #23 OR #24 OR #25 OR #26 OR #27 OR #28
30. #14 AND #29
    Filters: English, 2012-2022
    Search = 1,769

CINAHL

1. (MH "Spinal Stenosis")
2. (MH "Musculoskeletal Pain")
3. (MH "Shoulder Pain")
4. (MH "Low Back Pain")
5. (MH "Neck Pain")
6. (MH "Migraine")
7. (MH "Headache")
8. (MH "Chiropractic")
9. (MH "Manipulation, Chiropractic")
10. (MH "Manipulation, Orthopedic")
11. TX chiropractic
12. TX chiropractor
13. TX chiropractor
14. S1 OR S2 OR S3 OR S4 OR S5 OR S6 OR S7 OR S8 OR S9 OR S10 OR S11 OR S12 OR S13
15. (MH "Practice Guidelines")
16. TX "clinical practice guideline"
17. (MH "Quality of Health Care")
18. (MH "Quality Assurance")
19. (MH "Benchmarking")
20. TX "quality indicators"
21. (MH "Quality Improvement")
22. TX "standard of care"
23. (MH "Quality Control (Technology)")
24. (MH "Quality of Care Research")
25. TX "best-practice recommendations"
26. S15 OR S16 OR S17 OR S18 OR S19 OR S20 OR S21 OR S22 OR S23 OR S24 OR S25
27. S14 AND S26

Limiters - Published Date: 20120101-20221231; English Language; Research Article; Exclude MEDLINE records; Ebook exclude collection
Search = 585

Index to Chiropractic Literature

1. Subject:\"spinal stenosis\"
2. Subject:\"Musculoskeletal Pain\"
3. Subject:\"shoulder pain\"
4. Subject:\"low back pain\"
5. Subject:\"neck pain\"

6. Subject:\"migraine disorders\"
7. Subject:headache
8. Subject:chiropractic
9. Subject:\"Manipulation, Chiropractic\"
10. Subject:\"Manipulation, Orthopedic\"
11. All Fields:chiropractic
12. All Fields:chiropractor
13. All Fields:\"spine pain\"
14. All Fields:\"spinal pain\"
15. S1 OR S2 OR S3 OR S4 OR S5 OR S6 OR S7 OR S8 OR S9 OR S10 OR S11 OR S12 OR S13 OR S14
16. All Fields:\"clinical practice guideline\"
17. Subject:\"Practice Guidelines as Topic\"
18. Subject:guidelines
19. Subject:\"Guidelines as Topic\"
20. All Fields:standards
21. Subject:\"Quality of Health Care\"
22. Subject:\"Quality Assurance, Health Care\"
23. Subject:Benchmarking
24. Subject:\"Quality Indicators, Health Care\"
25. Subject:\"Quality Improvement\"
26. Subject:\"Quality Control\"
27. Subject:\"Standard of Care\"
28. All Fields:\"best-practice recommendations\"
29. S16 OR S17 OR S18 OR S19 OR S20 OR S21 OR S22 OR S23 OR S24 OR S25 OR S26 OR S27 OR S28
30. S15 AND S29
    Filters: Year: from 2012 to 2022, Peer Review only
    Search = 183

2,537 prior to duplicates removed: Pubmed = 1,769; CINAHL = 585; Index to Chiropractic Literature = 183.
