# Supplementary material for: Developing an initial set of quality indicators for chiropractic care: a scoping review
Source: BMC Health Serv Res. 2024 Jan 12;24:65. doi: 10.1186/s12913-024-10561-8 (PMC10785553; doi:10.1186/s12913-024-10561-8)
Supplement: Supplementary file 2 — Additional file 2. Search validation list. [file 12913_2024_10561_MOESM2_ESM.pdf]

Search validation list

List of known articles likely to meet most eligibility criteria and which should be included in search results.(1–24)

1. Blanchette MA, Mior S, Thistle S, Stuber K. Developing key performance indicators for the Canadian chiropractic profession: a modified Delphi study. Chiropr Man Ther. 2022 Dec;30(1):31.

2. Bryans R, Decina P, Descarreaux M, Duranleau M, Marcoux H, Potter B, et al. Evidence-Based Guidelines for the Chiropractic Treatment of Adults With Neck Pain. J Manipulative Physiol Ther. 2014 Jan;37(1):42–63.

3. Bussières AE, Stewart G, Al-Zoubi F, Decina P, Descarreaux M, Hayden J, et al. The Treatment of Neck Pain–Associated Disorders and Whiplash-Associated Disorders: A Clinical Practice Guideline. J Manipulative Physiol Ther. 2016 Oct;39(8):523-564.e27.

4. Bussières AE, Stewart G, Al-Zoubi F, Decina P, Descarreaux M, Haskett D, et al. Spinal Manipulative Therapy and Other Conservative Treatments for Low Back Pain: A Guideline From the Canadian Chiropractic Guideline Initiative. J Manipulative Physiol Ther. 2018 May;41(4):265–93.

5. Bussières A, Cancelliere C, Ammendolia C, Comer CM, Zoubi FA, Châtillon CE, et al. Non-Surgical Interventions for Lumbar Spinal Stenosis Leading To Neurogenic Claudication: A Clinical Practice Guideline. J Pain. 2021 Sep;22(9):1015–39.

6. Comer C, Ammendolia C, Battié MC, Bussières A, Fairbank J, Haig A, et al. Consensus on a standardised treatment pathway algorithm for lumbar spinal stenosis: an international Delphi study. BMC Musculoskelet Disord. 2022 Dec;23(1):550.

7. Côté P, Wong JJ, Sutton D, Shearer HM, Mior S, Randhawa K, et al. Management of neck pain and associated disorders: A clinical practice guideline from the Ontario Protocol for Traffic Injury Management (OPTIMa) Collaboration. Eur Spine J. 2016 Jul;25(7):2000–22.

8. Côté P, Yu H, Shearer HM, Randhawa K, Wong JJ, Mior S, et al. Non-pharmacological management of persistent headaches associated with neck pain: A clinical practice guideline from the Ontario protocol for traffic injury management (OPTIMa) collaboration. Eur J Pain Lond Engl. 2019 Jul;23(6):1051–70.

9. Globe G, Farabaugh RJ, Hawk C, Morris CE, Baker G, Whalen WM, et al. Clinical Practice Guideline: Chiropractic Care for Low Back Pain. J Manipulative Physiol Ther. 2016 Jan;39(1):1–22.

10. Hawk C, Schneider M, Evans MW, Redwood D. Consensus Process to Develop a Best-Practice Document on the Role of Chiropractic Care in Health Promotion, Disease Prevention, and Wellness. J Manipulative Physiol Ther. 2012 Sep;35(7):556–67.

11. Hawk C, Schneider MJ, Vallone S, Hewitt EG. Best Practices for Chiropractic Care of Children: A Consensus Update. J Manipulative Physiol Ther. 2016 Mar;39(3):158–68.

12. Hawk C, Schneider MJ, Haas M, Katz P, Dougherty P, Gleberzon B, et al. Best Practices for Chiropractic Care for Older Adults: A Systematic Review and Consensus Update. J Manipulative Physiol Ther. 2017 May;40(4):217–29.

13. Hawk C, Whalen W, Farabaugh RJ, Daniels CJ, Minkalis AL, Taylor DN, et al. Best Practices for Chiropractic Management of Patients with Chronic Musculoskeletal Pain: A Clinical Practice Guideline. J Altern Complement Med. 2020 Oct 1;26(10):884–901.

14. Hawk C, Amorin-Woods L, Evans MW , Jr, Whedon JM, Daniels CJ, Williams RD , Jr, et al. The Role of Chiropractic Care in Providing Health Promotion and Clinical Preventive Services for Adult Patients with Musculoskeletal Pain: A Clinical Practice Guideline. J Altern Complement Med N Y N. 2021 Oct;27(10):850–67.

15. Kjaer P, Kongsted A, Hartvigsen J, Isenberg-Jørgensen A, Schiøttz-Christensen B, Søborg B, et al. National clinical guidelines for non-surgical treatment of patients with recent onset neck pain or cervical radiculopathy. Eur Spine J. 2017 Sep;26(9):2242–57.

16. Lin I, Wiles L, Waller R, Goucke R, Nagree Y, Gibberd M, et al. What does best practice care for musculoskeletal pain look like? Eleven consistent recommendations from high-quality clinical practice guidelines: systematic review. Br J Sports Med. 2020 Jan;54(2):79–86.

17. Nijs J. Low Back Pain: Guidelines for the ClinicalClassification of Predominant Neuropathic,Nociceptive, or Central Sensitization Pain. Pain Physician. 2015 May 14;3;18(3;5):E333–46.

18. Qaseem A, Wilt TJ, McLean RM, Forciea MA. Noninvasive Treatments for Acute, Subacute, and Chronic Low Back Pain: A Clinical Practice Guideline From the American College of Physicians. Ann Intern Med. 2017 Apr 4;166(7):514–30.

19. Rousing R, Jensen RK, Fruensgaard S, Strøm J, Brøgger HA, Degn JDM, et al. Danish national clinical guidelines for surgical and nonsurgical treatment of patients with lumbar spinal stenosis. Eur Spine J. 2019 Jun;28(6):1386–96.

20. Stochkendahl MJ, Kjaer P, Hartvigsen J, Kongsted A, Aaboe J, Andersen M, et al. National Clinical Guidelines for non-surgical treatment of patients with recent onset low back pain or lumbar radiculopathy. Eur Spine J. 2018 Jan;27(1):60–75.

21. Weis CA, Pohlman K, Barrett J, Clinton S, da Silva-Oolup S, Draper C, et al. Best-Practice Recommendations for Chiropractic Care for Pregnant and Postpartum Patients: Results of a Consensus Process. J Manipulative Physiol Ther. 2021 Nov;S0161475421000361.

22. Whalen W, Farabaugh RJ, Hawk C, Minkalis AL, Lauretti W, Crivelli LS, et al. Best-Practice Recommendations for Chiropractic Management of Patients With Neck Pain. J Manipulative Physiol Ther. 2019 Nov;42(9):635–50.

23. Wong JJ, Côté P, Sutton DA, Randhawa K, Yu H, Varatharajan S, et al. Clinical practice guidelines for the noninvasive management of low back pain: A systematic review by the Ontario Protocol for Traffic Injury Management (OPTIMa) Collaboration. Eur J Pain. 2017 Feb;21(2):201–16.

24. Yu H, Côté P, Wong JJ, Shearer HM, Mior S, Cancelliere C, et al. Noninvasive management of soft tissue disorders of the shoulder: A clinical practice guideline from the Ontario Protocol for Traffic Injury Management (OPTIMa) collaboration. Eur J Pain. 2021 Sep;25(8):1644–67.
