# Supplementary material for: Developing an initial set of quality indicators for chiropractic care: a scoping review
Source: BMC Health Serv Res. 2024 Jan 12;24:65. doi: 10.1186/s12913-024-10561-8 (PMC10785553; doi:10.1186/s12913-024-10561-8)
Supplement: Supplementary file 3 — Additional file 3. Quality indicator 2nd level review checklist. [file 12913_2024_10561_MOESM3_ESM.pdf]

Quality indicator 2nd level review checklist

**Note: For all No and Unclear responses, document reasons/questions in the reviewer notes field in the corresponding spreadsheet*

1. Identify source document. ☐
2. Title: Does the title match the description? ☐ Y ☐ N ☐ Unclear
3. Is condition category consistent with source document? ☐ Y ☐ N ☐ Unclear
   **Note: Some abstracted indicators were genericized because they are applicable to multiple problems, such as the need for an exam regardless of problem (e.g., headache, neck pain, etc.)*
4. Is the Donabedian category appropriate? ☐ Y ☐ N ☐ Unclear
5. Is the IOM domain appropriate? ☐ Y ☐ N ☐ Unclear
6. Is the evidence level appropriate? ☐ Y ☐ N ☐ Unclear
   **Note: when references supporting an abstracted indicator are not listed in the source document, the default grading is 5. When references are available, review to determine grading level*
7. Is the metric understandable? ☐ Y ☐ N ☐ Unclear
8. Does the metric appear to measure the intended indicator? ☐ Y ☐ N ☐ Unclear
   **Note: The metric should measure what is described in the title and description*
9. Does the indicator meet all 5 SMART criteria? ☐ Y ☐ N ☐ Unclear
10. Is the indicator distinct from others from the same source? ☐ Y ☐ N ☐ Unclear
